# Multi-resolution independent component analysis for high-performance tumor classification and biomarker discovery

**DOI:** 10.1186/1471-2105-12-S1-S7

**Published:** 2011-02-15

**Authors:** Henry Han, Xiao-Li Li

**Affiliations:** 1 Center for Computational Medicine and Bioinformatics, University of Michigan, Ann Arbor MI 48109, USA; 2 Departament of Mathematics and Bioinformatics, Eastern Michigan University, Ypsilanti MI 48197, USA; 3 Institute for Infocomm Research, Agency for Science, Technology and Research (A*STAR), Singapore 138632

## Abstract

**Background:**

Although high-throughput microarray based molecular diagnostic technologies show a great promise in cancer diagnosis, it is still far from a clinical application due to its low and instable sensitivities and specificities in cancer molecular pattern recognition. In fact, high-dimensional and heterogeneous tumor profiles challenge current machine learning methodologies for its small number of samples and large or even huge number of variables (genes). This naturally calls for the use of an effective feature selection in microarray data classification.

**Methods:**

We propose a novel feature selection method: multi-resolution independent component analysis (MICA) for large-scale gene expression data. This method overcomes the weak points of the widely used transform-based feature selection methods such as principal component analysis (PCA), independent component analysis (ICA), and nonnegative matrix factorization (NMF) by avoiding their global feature-selection mechanism. In addition to demonstrating the effectiveness of the multi-resolution independent component analysis in meaningful biomarker discovery, we present a multi-resolution independent component analysis based support vector machines (MICA-SVM) and linear discriminant analysis (MICA-LDA) to attain high-performance classifications in low-dimensional spaces.

**Results:**

We have demonstrated the superiority and stability of our algorithms by performing comprehensive experimental comparisons with nine state-of-the-art algorithms on six high-dimensional heterogeneous profiles under cross validations. Our classification algorithms, especially, MICA-SVM, not only accomplish clinical or near-clinical level sensitivities and specificities, but also show strong performance stability over its peers in classification. Software that implements the major algorithm and data sets on which this paper focuses are freely available at https://sites.google.com/site/heyaumapbc2011/.

**Conclusions:**

This work suggests a new direction to accelerate microarray technologies into a clinical routine through building a high-performance classifier to attain clinical-level sensitivities and specificities by treating an input profile as a ‘profile-biomarker’. The multi-resolution data analysis based redundant global feature suppressing and effective local feature extraction also have a positive impact on large scale ‘omics’ data mining.

## Background

With the rapid developments in genomics, high-throughput microarray pattern analysis shows a great potential in cancer diagnosis for its efficiency and cost-effectiveness [1]. However, such a promising technology remains an important research field rather than an applicable clinical-routine. Aside intrinsic factors from microarray profiling technologies, a key issue preventing it from becoming a clinical paradigm is that the relatively low even poor sensitivities and specificities obtained from current pattern recognition methodologies are inadequate to provide a robust clinical support. Moreover, some pattern classification methods may perform reasonably well in some data sets but fail badly in others. Although there is an urgent need in clinical cancer research to develop high-performance pattern recognition methods in gene expression analysis, it is still a challenge in machine learning to attain high-accuracy classification for the special characteristics of gene expression profiles.

A gene expression profile can be represented by a *p×n* matrix after preprocessing, each column of which represents gene expression values of all biological samples at a gene; each row of which represents gene expression values of a single biological sample across a genome. The total number of genes is in the order of 10^3^*~*10^4^, and the total number of biological samples is on the magnitude of tens or hundreds. Since the number of variables (genes) is much greater than the number of samples (observations), some traditional pattern recognition methods (e.g., fisher discriminant analysis) may have instable solutions and lead to a low or poor classification performance. Alternatively, although there are a large number of genes in a profile, only a small portion of them have meaningful contributions to data variations. In addition, the high-dimensional data are not noise-free because preprocessing algorithms may not remove systematic noise contained in raw data completely. Obviously, the data redundancy and noise may inevitably affect the discriminative power of the classification algorithms applied to microarray data.

It is clear that feature selection play a critical role in gene expression analysis to decrease dimensionalities, remove noise, and extract meaningful features before performing classification. Feature selection algorithms usually can be categorized into three types: statistical test-based (e.g., two-sample t-tests), wrapper-based (e.g., SVM-based wrappers) [2], and transform-based feature selections. The transform-based feature selection may be mostly used data reduction techniques for their popularity and efficiency. They include principal component analysis (PCA) [3], independent component analysis (ICA) [4], nonnegative matrix factorization (NMF) [5,6], etc, and their different extensions [7,8].

However, these transform-based feature selection algorithms are generally good at selecting global features instead of local features. The global and local features contribute to the global and local characteristics of data and interpret global and local behavior of data respectively. Statistically, the global features consist of high-frequency signals and the local features consist of low-frequency signals. Unlike the global features, the local features are difficult to extract for most feature-selection algorithms, because the low-frequency signals have a lower likelihood to get involved in inferring the ‘new’ low-dimensional data, which are generally the linear combinations of all input variables, than the high-frequency signals. Finally, the low dimensional data obtained from the traditional feature selection methods may miss some local data characteristics described by the local features. For example, PCA is by-nature a global feature selection algorithm: each principal component contains some levels of global characteristics of data and receives contributions from all input variables in the linear combinations. In addition, changes in one variable will inevitably affect all loading vectors globally. However, local features may be a key to attaining high-performance gene expression pattern classification for its subtle data behavior capturing. For example, some benign tumor samples may display very similar global characteristics with malignant tumor samples but with different local characteristics. To attain high-performance diagnosis, it is essential to capture local data characteristics to distinguish these samples with similar global characteristics.

The main reason for these algorithms’ global-feature selection mechanism is because they all are single-resolution feature selection methods, where all features are indistinguishably displayed in a single-resolution despite the nature of their frequencies. It inevitably causes global features more likely to be selected than local features and prevents effective local data-characteristics capturing. Mathematically, all variables of the input data are involved in the linear combinations to compute principal components in PCA, independent components in ICA, and basis vectors in NMF respectively. Such a global feature selection mechanism will prevent high-accuracy genomic pattern recognition in the following classification because only the features interpreting global characteristics are involved in training a learning machine (e.g., SVM). The redundant global features may inevitably decrease the generalization of the learning machine and increase the risk of misclassifications or over-fitting. Finally, the learning machines integrated with the global feature-selection algorithms will display instabilities in classifications.

To avoid the global feature selection mechanism, it is desirable to distinguish (e.g., sort) features according to their frequencies rather than treat them uniformly, which makes the high-frequency signals dominate the feature selection and the low-frequency signals lose opportunities. A discrete wavelet transform (DWT) [9] can hierarchically organize data in a multi-resolution way by low and high pass filters. The low (high)-pass filters only pass low (high)-frequency signals but attenuate signals with frequencies higher (lower) than a cutoff frequency. Finally, the DWT coefficients at the coarse levels capture global features of the input signals and the coefficients at the fine levels capture local features of the signals, i.e., the low-frequency and high-frequency signals are represented by coefficients in the coarse and fine resolutions respectively. Obviously, the global feature selection mechanism can be relatively easy to overcome after such a ‘multi-resolution feature separation’, by selectively extracting local features and filtering redundant global features.

In this study, we propose a novel multi-resolution independent component analysis (MICA) to conduct effective feature selections for high dimensional heterogeneous gene expression data. Then, a multi-resolution independent component analysis based support vector machines (MICA-SVM) are proposed to achieve a high-performance gene expression pattern prediction. We demonstrate its superiority and stability by comparing it with existing state-of-the-art peers on six heterogeneous microarray profiles, in addition to extending MICA to linear discriminant analysis (MICA-LDA). We also develop a MICA-based filter-wrapper biomarker discovery algorithm to further demonstrate the novel feature selection algorithm’s effectiveness in biomarker capturing. Finally, we discuss potential extensions on the multi-resolution independent component analysis in microarray based molecular diagnosis and conclude this paper.

## Methods

Multi-resolution independent component analysis is based on the discrete wavelet transform (DWT) and independent component analysis (ICA). A discrete wavelet transform decomposes input data in a multi-resolution form by using a wavelet and scaling function. The coefficients at the coarse and fine levels describe the global and local behavior of data respectively. Mathematically, DWT is equivalent to multiplying the input data by a set of orthogonal matrices block-wisely. On the other hand, ICA seeks to represent input data as the linear combination of a set of statistically independent components by minimizing their mutual information. Theoretically, it is equivalent to inverting the central limit theorem (CLT) by searching maximally non-normal projections of the original data distribution. More information about the DWT and ICA methods can be found in [4,9].

## Multi-resolution independent component analysis

The goal of the multi-resolution independent component analysis is to seek the statistically independent genomic patterns from a meta-profile computed by suppressing the coarse level coefficients (global features) and maintaining the fine level coefficients (local features) in the DWT of an input profile. As an approximation of the high dimensional input profile, the derived meta-profile captures almost all local features and keeps the most important global features. Unlike independent components in the classic ICA that are mainly retrieved from the global features for their high frequencies, the independent components calculated by our proposed MICA method are statistically independent signals, which contain contributions from almost all local features and the most important global features. As such, the latter is more representative in revealing the latent data structure than the former. Moreover, the redundant global feature suppressing brings MICA an automatic de-noising mechanism: since the coarse level coefficients (e.g., the first level coefficients) in DWT generally contain “contributions” from noise, suppressing coarse level coefficients not only filters unnecessary global features but also removes the noise. The MICA algorithm can be described as following steps.

### 1). Wavelet transforms

Given a gene expression profile with *p* samples across *n* genes , MICA conducts a *L*-level discrete wavelet transform for each sample to obtain a sequence of detail coefficient matrices  and an approximation coefficient matrix , i.e., , where .

### 2). Feature selection

A level threshold  is selected to suppress redundant global features and maintain local features as follows. If , 1) conduct principal component analysis for *D_j_* to obtain its PC matrix:  and the corresponding score matrix  . 2) reconstruct the original *D_j_* by using the first loading vector *u_1_* in the PC matrix as , where  is a vector containing all ‘1’s. If , reconstruct and update each detail coefficient matrix *D_j_* by using the loading vectors  with the 100% explained variance percentage and their corresponding vectors in the score matrix: . The explained variance percentage is the ratio between the accumulative variance from the selected data and the total data variance. For example, the explained variance percentage *ρ_r_* from those first *r* loading vectors is defined as , where *λ_i_* is the data variance from the *i_^th^_* loading vector. In the implementation, this step can be ‘lazily’ simplified as: keep all detail coefficient matrices  intact to save computing resources.

### 3). Inverse discrete wavelet transforms

Conduct the corresponding inverse discrete wavelet transform using the updated coefficient matrices  to get the meta-profile of *X*:, i.e., .

### 4). Independent component analysis

Conduct the classic independent component analysis for X^*^ to obtain independent components and the mixing matrix: , where , and .

### 5). Subspace decomposition

The meta-profile X^*^ is the approximation of the original profile *X* by removing the redundant global features and retaining almost all local features by selecting features on behalf of their frequencies. It is easy to decompose each sample in the subspace spanned by all independent components . Each independent component is a basis in the subspace., i.e., , where the mixing matrix is , and the independent component matrix is . In other words, each sample can be represented as , where the meta-sample *a_i_* is the *i^th^* row of the mixing matrix recording the coordinate values of the sample *x_i_* in the subspace. As a low dimensional vector, the meta-sample *a_i_* retains almost all local features and the most outstanding global features of the original high-dimensional sample *x_i_*. Thus it can be called as a data-locality preserved prototype of *x_i_*.

**Figure 1:**
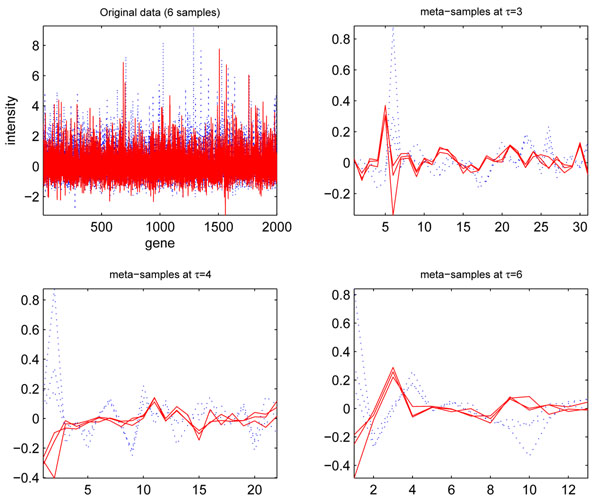
**Meta-samples constructed from MICA for six original samples ('*breast_1 data*')**. Meta-samples constructed from multi-resolution independent component analysis for six original samples (three controls and three cancers) in the breast_1 data at the three levels thresholds: *τ =*3,4,6 with a wavelet ‘*db8*’. The low-dimensional meta-samples separate two types of samples clearly in visualization.

Figure 1 visualizes three controls and cancers of the ‘breast_1’ data (see Table 1 for more information) and their meta-samples obtained from MICA at *τ =* 3,4,6 with a *Daubechies* family wavelet ‘*db8*’, where the control and cancer samples are indicated by *red* and *blue* lines respectively. Interestingly, extracted local features and selected important global features make two types of samples display two distinct prototypes in the low-dimension subspace. With the increase of the level thresholds, the two groups of prototypes tend to show more capabilities to separate cancer and control samples. Moreover, two types of meta-samples demonstrate a “self-clustering” mechanism in that the meta-samples belonging to the same type show close spatial proximities. Obviously, the clear sample separation information conveyed by the self-clustering mechanism of the meta-samples is almost impossible to obtain from the original high-dimensional data directly, and the key discriminative features captured by our proposed MICA method would be able to facilitate the subsequent classification step and contribute to high-accuracy disease diagnosis.

**Table 1:** Six gene-expression microarray profiles

Dataset	#Genes	#Samples	Technology
Stroma	18995	13 inflammatory breast cancers (‘*ibc*’) + 34 non-inflammatory breast cancers (‘*non-ibc*’)	Oligonucleotide
Breast_1	2000	53 controls + 163 cancers	Oligonucleotide
Prostate	12600	59 controls + 77 cancers	Oligonucleotide
Glioma	12625	28 glioblastomas + 22 anaplastic oligodendrogliomas	Oligonucleotide
HCC	7129	20 early intrahepatic recurrence + 40 non-early intrahepatic recurrence	Oligonucleotide
Breast_2	24188	46 samples with distant metastasis within 5 year + 51 samples remain disease-free within 5 years	cDNA

### Multi-resolution Independent component analysis based support vector machines (MICA-SVM)

The MICA-based support vector machines apply the classic support vector machines (C-SVM) [10] to the meta-samples to gain classification information in a low-dimensional space. Unlike the traditional SVM that builds a maximum margin hyper-plane in the original data space ℝ*^n^* where *n~*10^3^-10^4^, MICA-SVM separates biological samples by constructing the maximum margin hyperplane in the spanned subspace  where *k ≤ p ~* 10^2^, using the meta-samples. If we assume the number of support vectors *N_s_* is much less than the training points *l*, the time complexity of the MICA-SVM is , which is much lower than that of the classic SVM , provided the same number of training points and support vectors. We briefly describe the MICA-SVM classifier for binary classification. Given a training dataset , and sample class type information , where , a meta-dataset  is computed by using the multi-resolution independent component analysis. Then, a maximum margin hyper-plane:  is constructed to separate the '+1' (‘cancer’) and '-1' (‘control’) types of meta-samples. It is equivalent to solving the following quadratic programming problem,(1)

A way to solve (1) is through its Lagrangian dual that is also a quadratic programming problem, where  are dual variables of the primal variables *w* and *b*.(2)

The normal of the maximum-margin hyperplane is calculated as . The decision rule  is used to determine the class type of a testing sample *x′*, where  are the corresponding meta-samples of samples  , computed from MICA respectively. The function  is a SVM kernel function that maps these meta-samples into a same-dimensional or high-dimensional feature space. In this work, we only focus on the linear kernel for its simplicity and efficiency in microarray pattern classifications. We will point out in the discussion section that most SVM-based learning machines would encounter overfitting under the standard Gaussian kernel (‘*rbf*’: radial basis function kernels).

## Results

We have performed extensive experiments using six publicly available gene expression microarray profiles consisting of five oligonucleotide profiles [11-15] and one cDNA profile [16], in the experiment. Table 1 includes their detailed information. These profiles are heterogeneous data generated from different experimental conditions, different profiling technologies, or even processed by different preprocessing algorithms. For example, the stroma, prostate, glioma, and HCC data only go through basic log2 transforms while the breast_1 data is a dataset obtained by conducting two-sample t-tests from an original dataset going through delicate normalizations [12].

### Cross validations

To address our algorithm’s superiority and reproducibility, we compare it with six comparison algorithms in terms of average classification rates, sensitivities, and specificities under the *k*-fold (*k*=10) and 100-trial of 50% holdout cross validations. The classification accuracy in the *i^th^* classification is the ratio of the correctly classified testing samples over total testing samples: , and the sensitivity and specificity are defined as the ratios:  respectively, where *tp* (*tn*) is the number of positive (negative) targets correctly classified, and *fp (fn)* is the number of negative (positive) targets incorrectly classified respectively. In the 100-trial of 50% holdout cross validation (HOCV), all samples in the data set are pooled together and randomly divided into half to get training and testing data. Such a partition is repeated 100 times to get 100 sets of training and testing datasets. In the *k*-fold cross validation, an input dataset is partitioned into *k* disjoint equal or approximately equal proportions. One proportion is used for testing and the other *k-1* proportions are used for training alternatively in the total *k* rounds of classifications. Compared with pre-specified training or testing data, the cross validations can decrease potential biases in algorithm performance evaluations.

### Six comparison algorithms

The existing six comparison algorithms can be categorized into two types. The first type consists of standard support vector machines (SVM) [10] and linear discriminant analysis (LDA) [17], both of which are state-of-the-art classification algorithms. Especially, SVM is widely employed in gene expression pattern recognition for its popularity. The second type consists of four methods embedding transform-based feature selections in SVM and LDA: they are support vector machines with principal component analysis/independent component analysis/ nonnegative matrix factorization, and linear discriminant analysis with principal component analysis. We refer them as PCA-SVM, ICA-SVM, NMF-SVM, and PCA-LDA conveniently and their related implementation information can be found in Additional file 1.

We employ the wavelet ‘*db8*’ to conduct a 12-level discrete wavelet transform for each data set, and select a level threshold *τ =* 3 in MICA for all profiles. Although not an optimal level threshold for all data, it guarantees automatic de-noising and ‘fair’ algorithm comparisons. Moreover, we have found that the meta-samples obtained from MICA at *τ =* 3 can clearly distinguish two types of samples. Although other level threshold selections may be possible, any too ‘coarse’ (e.g.*τ =* 1) or too ‘fine’ (e.g.*τ ≥* 9) level threshold selection may miss some important global or local features and affect following classifications.

Table 2 and Table 3 illustrate the average performance of the seven algorithms in terms of the classification rates, sensitivities, specificities and their standard deviations under the two types of cross validations respectively. The results of LDA are not included in the two tables for its worst performance. Similarly, the NMF-SVM and ICA-SVM algorithms are excluded from Table 3 for their relatively low performance and high instabilities. Clearly, the proposed MICA-SVM algorithm demonstrates *exceptionally* leading advantages over its peers in the three classification performance statistics for all datasets. For example, it achieves 98.26%, 99.04%, 99.69%, 98.76%, 98.30% and 97.23% average classification rates on the stroma, breast_1, prostate, glioma, HCC, and breast_2 data respectively under the 100 trials of 50% HOCV. In addition, MICA-SVM achieves 98.00%, 99.52%, 100.0%, 100.0%, 100.0%, and 99.00% for the stroma, breast_1, prostate, glioma, HCC, and breast_2 data respectively under the 10-fold CV. All these results indicate that MICA can effectively capture global/local features as well as eliminate the noisy features so that SVM can perform *significantly* better than the state-of-the-arts. Furthermore, unlike the other methods that display instabilities in classifications, our proposed MICA-SVM algorithm demonstrates a strong stability in attaining high-accuracy detections for all profiles. This observation is also supported by its lower standard deviations of the three classification measures than those of the others.

**Table 2:** Algorithm average performance comparisons (100 trials of 50% HOCV)

Dataset	Avg. classification rate ±std (%)	Avg. sensitivity ± std (%)	Avg. specificity ± std (%)
**Stroma**			
*mica-svm*	**98.26±02.25**	**100.0±00.00**	**93.89±08.11**
*svm*	73.83±07.02	92.87±06.58	25.45±15.92
*pca-svm*	71.83±06.78	90.20±08.66	25.62±16.48
*ica-svm*	71.48±06.78	90.04±09.05	25.06±17.87
*nmf-svm*	68.39±08.67	86.30±11.93	23.69±11.93
*pca-lda*	71.35±06.97	89.12±09.15	26.69±17.05
**Breast_1**			
*mica-svm*	**99.04±00.99**	**99.49±01.18**	**97.73±02.95**
*svm*	86.40±02.87	92.43±02.76	68.78±11.53
*pca-svm*	86.19±02.97	92.79±02.68	66.85±11.77
*ica-svm*	86.27±02.99	92.80±02.82	67.11±12.37
*nmf-svm*	85.44±02.42	93.52±02.91	61.29±09.10
*pca-lda*	86.25±02.89	92.43±02.83	68.15±11.95
**Prostate**			
*mica-svm*	**99.69±00.67**	**99.88±00.64**	**99.44±01.38**
*svm*	91.16±02.58	89.53±04.53	93.42±04.57
*pca-svm*	90.76±02.65	89.18±04.60	92.94±04.76
*ica-svm*	61.43±08.54	78.75±23.15	41.09±28.88
*nmf-svm*	71.03± 07.27	88.48±07.17	49.84±19.33
*pca-lda*	90.47±03.46	89.46±04.81	91.87±05.76
**Glioma**			
*mica-svm*	**98.76±02.03**	**98.89±02.90**	**98.82±02.89**
*svm*	74.00±07.51	68.19±12.71	79.45±11.30
*pca-svm*	72.60±06.81	69.05±14.38	76.25±11.69
*ica-svm*	47.20±08.79	25.24±29.21	69.61±29.55
*nmf-svm*	74.40±08.04	74.53±11.10	74.19±13.53
*pca-lda*	73.96±07.02	68.38±12.41	79.18±12.39
**HCC**			
*mica-svm*	**98.30±02.30**	**99.23±02.02**	**96.97±06.05**
*svm*	61.53±07.75	75.04±12.40	37.15±18.27
*pca-svm*	60.93±07.90	72.82±14.19	39.53±17.70
*ica-svm*	58.73±07.29	72.37±12.51	29.72±15.93
*nmf-svm*	61.30±08.91	71.17±13.47	43.47±16.67
*pca-lda*	61.07±07.58	74.15±12.40	37.15±17.08
**Breast_2**			
*mica-svm*	**97.23±03.20**	**97.79±03.90**	**96.93±05.17**
*svm*	63.04±05.48	65.81±11.20	61.59±13.17
*pca-svm*	62.29±05.54	66.86±12.09	59.00±13.72
*ica-svm*	62.27±05.59	67.39±11.51	58.28±13.81
*nmf-svm*	62.77±06.60	66.92±10.68	59.57±13.69
*pca-lda*	62.54±05.48	66.94±11.90	59.39±13.25

**Table 3:** Algorithm average performance comparisons (10-fold CV)

Dataset	Avg. classification rate ± std (%)	Avg. sensitivity ± std (%)	Avg. specificity ± std (%)
**Stroma**			
*mica-svm*	**98.00 ± 06.32**	**100.0 ± 00.00**	**95.00 ± 15.81**
*pca-lda*	71.83 ± 11.15	94.17 ± 12.45	15.00 ± 33.75
*svm*	74.83 ± 19.76	90.83 ± 14.93	35.00 ± 47.43
*pca-svm*	71.00 ± 16.47	91.67 ± 18.00	15.00 ± 33.75
**Breast_1**			
*mica-svm*	**99.52 ± 01.51**	**100.0 ± 00.00**	**98.00 ± 06.32**
*pca-lda*	88.51 ± 06.10	90.88 ± 07.60	81.00 ± 15.56
*svm*	87.49 ± 06.85	91.91 ± 08.37	75.00 ± 22.62
*pca-svm*	88.00 ± 04.99	91.47 ± 05.81	77.00 ± 15.27
**Prostate**			
*mica-svm*	**100.0 ± 00.00**	**100.0 ± 00.00**	**100.0 ± 00.00**
*pca-lda*	93.29 ± 05.50	90.71 ± 08.74	96.67 ± 07.03
*svm*	94.12 ± 05.84	92.32 ± 06.63	96.33 ± 07.77
*pca-svm*	93.35 ± 05.48	92.32 ± 08.87	95.00 ± 08.05
**Glioma**			
*mica-svm*	**100.0 ± 00.00**	**100.0 ± 00.00**	**100.0 ± 00.00**
*pca-lda*	76.33 ± 18.93	68.33 ± 36.39	81.67 ± 19.95
*svm*	75.67 ± 19.82	66.67 ± 33.33	81.67 ± 19.95
*pca-svm*	78.00 ± 17.98	68.33 ± 27.72	86.67 ± 17.21
**HCC**			
*mica-svm*	**100.0 ± 00.00**	**100.0 ± 00.00**	**100.0 ± 00.00**
*pca-lda*	68.33 ± 14.59	80.00 ± 15.81	45.00 ± 43.78
*svm*	71.67 ± 15.81	82.50 ± 16.87	50.00 ± 33.33
*pca-svm*	63.33 ± 17.21	77.50 ± 14.19	35.00 ± 33.75
**Breast_2**			
*mica-svm*	**99.00 ± 03.16**	**100.0 ± 00.00**	**98.00 ± 06.32**
*pca-lda*	62.77 ± 20.39	59.33 ± 19.74	66.50 ± 28.87
*svm*	67.61 ± 17.41	66.33 ± 25.26	69.00 ± 29.89
*pca-svm*	62.94 ± 13.09	60.67 ± 23.19	65.00 ± 22.36

**Figure 2:**
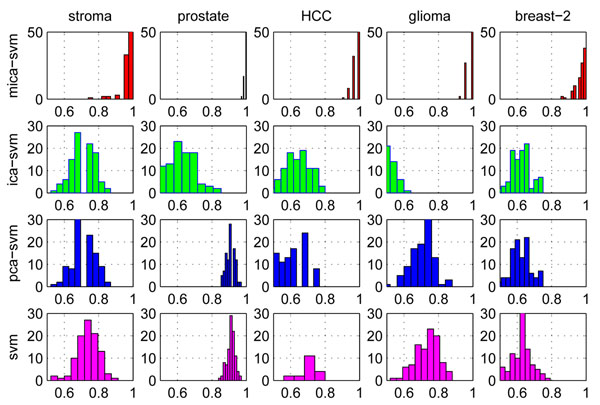
**Distributions of the classification rates of four algorithms on five profiles.** Distributions of the classification rates of four algorithms: MICA-SVM, ICA-SVM, PCA-SVM, and SVM on five profiles under the 100 trials of 50% holdout cross validations

**Figure 3:**
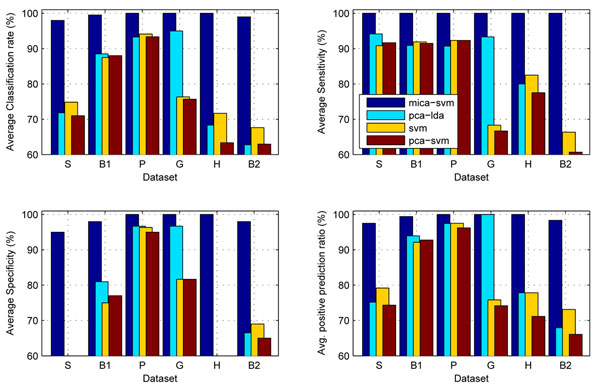
**Comparisons on the five algorithm performance on the six datasets under k-fold cross validations**. Comparisons on the five algorithm classification performance on the six datasets under k-fold cross validations. ‘S’ (*stroma*), ‘B1’ (*breast_1*), ‘P’ (*prostate*), ‘G (*glioma*)’‘H’ (HCC), and ‘B2’ (*breast_2*). The MICA-SVM algorithm demonstrated exceptional leading performance over the others

Figure 2 compares the distributions of the classification rates of the four algorithms on the other five profiles under the 100 trials of 50% HOCV. It is obvious that the distributions of classification rates, sensitivities and specificities of MICA-SVM on these data are significantly different from those of the other three peers. Moreover, it seems that there is no statistically significant improvement between SVM and its feature-selection based extensions: ICA-SVM, PCA-SVM, and NMF-SVM, because they achieved the same or slightly lower performance than the standard SVM. The reason for this is rooted in the global feature selection mechanisms of the PCA, ICA, and NMF methods: since biological samples may display very similar global-characteristics and different local-characteristics in their gene expressions, a classification algorithm (e.g., SVM) integrated with the global-feature selection methods will inevitably encounter difficulty in distinguishing these samples. Although extracted by different transform methods, the global features statistically have almost same level contributions to the pattern classifications of a data set. Moreover, the redundant global features brought by the global feature selection mechanism may be involved in the following SVM learning, which limits all the SVM extensions’ generalization and causes their instabilities in classification. However, the local feature capturing and redundant global feature suppressing mechanism in MICA not only attains much better performance than the standard SVM but also maintains algorithm stability in classification. Moreover, Figure 3 shows the MICA-SVM’s leading advantages over the other four peers on behalf of the average classification rates, sensitivities, specificities, and positive prediction ratios under the 10-fold cross validations. All the results directly demonstrate the superiority of MICA to the three general global feature selection algorithms.

### Multi-resolution independent component analysis based linear discriminant analysis

**Figure 4:**
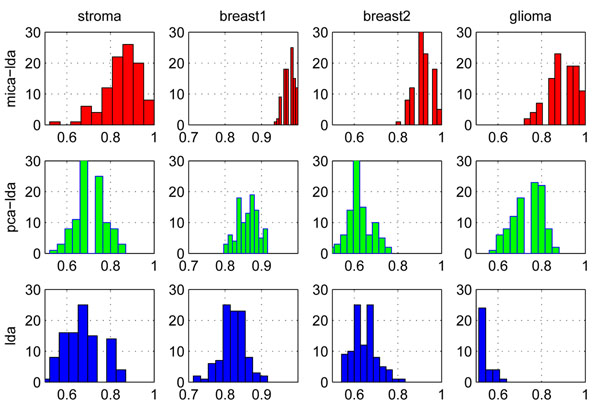
**Comparisons of the distributions of algorithm classification rates**. Comparisons of the distributions of classification rates of three algorithms on four profiles under the 100 trials of 50% HOCV. LDA classification rates < 50% on the glioma data are not showed in the visualization

We also apply MICA to linear discriminant analysis (LDA) to further explore its effectiveness. Similar to the MICA-SVM algorithm, the MICA-based linear discriminant analysis (MICA-LDA) applies the classic LDA to the meta-samples obtained from MICA to gain sample classifications (We skip the detailed algorithm description on MICA-LDA for the space constraint). The MICA-LDA algorithm’s performance on the six profiles can be found in the Additional file 2. To keep consistency with the previous experiments, we still employ the ‘*db8*’ wavelet and set the level threshold *τ =* 3 in MICA. Interestingly, the MICA-LDA classifier is only secondary to the MICA-SVM classifier: it outperforms the other comparison algorithms on the five datasets except the prostate data in terms of the average performance under the 100 trials of HOCV and 10-fold CV. This further indicates that MICA’s effective feature selection and its contribution to subsequent classification methods. Figure 4 compares the distribution of classification rates from the three LDA-based algorithms: MICA-LDA, PCA-LDA, and LDA on four data sets under the 100 trials of 50% HOCV. Interestingly, MICA-LDA obviously outperforms PCA-LDA and LDA by its right-skewed classification rate distributions. Although PCA-LDA also demonstrates classification advantages over LDA, MICA-LDA has attained much more impressive improvements than PCA-LDA. On the other hand, this also indicates that the multi-resolution independent component analysis is more effective in the feature selection than principal component analysis, which contributes directly to improving LDA classifier’s performance.

### Optimal level threshold selections

A remaining question is how to determine the optimal level threshold in MICA so that the following SVM classifier achieves best performance. We employ the condition number  of the independent component matrix *Z* in MICA to resolve it, where *S*_max_ and *S*_min_ are the maximum and minimum singular values of the matrix *Z* calculated from MICA. A smaller condition number indicates a more stable matrix that suggests a better status in global and local feature capturing. The level-threshold is counted ‘optimal’ if the condition number *δ* is the smallest. If the condition numbers from two level thresholds are same numerically, the lower level threshold (which is required to be > 1) is counted as the optimal one. For example, the smallest *δ* value is achieved at *τ =* 6 and *τ =* 7,8,9,10,11 respectively on the HCC data. We choose *τ =* 6 as the optimal threshold which is corresponding to the best average the average classification rate: 98.77% (STD: 2.26%) with average sensitivity: 99.44% (±2.11%) and specificity are 97.59% (±4.97%) respectively.

**Figure 5:**
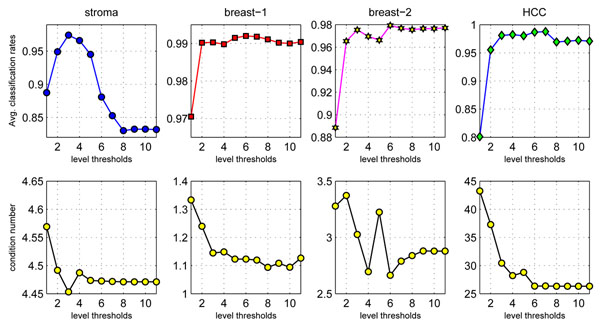
**Optimal threshold selections**. Average classification rates and corresponding condition numbers at 11 level thresholds on four profiles under 100 trials of 50% HOCV.

Figure 5 shows the MICA-SVM average classification rates and corresponding condition number *δ* values under the 100 trials of 50% HOCV on the ‘stroma’, ‘breast_1’, and ‘breast_2’, and ‘HCC’ data, as the level threshold values in MICA are selected from 1 to 11. Obviously, the optimal level threshold can be identified by finding the level threshold corresponding to the minimum condition number. Although the optimal threshold at *τ =* 8 corresponding to average classification rate 99.11% (±0.89%), which is slightly lower than the actual best average classification rate: 99.20% (±0.92%) achieved at *τ =* 6, it is ignorable due to possible numerical inaccuracy from the fixed point iteration in MICA. Furthermore, we have found that MICA-SVM has relatively low-level performance at too coarse level thresholds (e.g. *τ =* 1). Although *δ* values and MICA-SVM performance show some-level stability under some fine level thresholds, too fine level thresholds (e.g. *τ ≥* 8) may decrease classification performance on some data (e.g., stroma data). Also, the optimal level threshold selection method may bring some computing overhead in practical classification. In practice, we suggest the empirical level threshold as  for its relative robust performance and automatic de-noising property.

Although only wavelet ‘*db8*’ is employed in our experiments, there is no other specific requirement in MICA-SVM for a wavelet except it should be orthogonal. To compare effects of different wavelet selections on the algorithm performance, we select four family wavelets: ‘*db8*’, ‘*sym8*’, ‘*coif4*’, and ‘*bior4.4’*, in the classifications on the six profiles at the level threshold *τ =* 3. It seems that there is no obvious classification advantage from one wavelet over the other under the 10-fold CV, because the robust prior knowledge and less number of trials may have larger impact factors on the algorithm performance than a wavelet selection. However, we have found that the wavelet ‘*db8*’ show some advantages over the others under the 100 trials of 50% HOCV. In addition, it is interesting to see that the wavelets ‘*coif4*’ and ‘*sym8*’ have almost same-level performance, but the wavelet *‘bior4.4*’ has a relatively low performance for the six profiles.

We further demonstrate the superiority of MICA-SVM by comparing it with three state-of-the-art partial least square (PLS) based regression methods, which can be found in the Additional file 3. Moreover, we present a novel algorithm stability analysis for the seven classifications and show the advantages of the MICA-SVM and MICA-LDA algorithms over the others (Please see the Additional file 4 for details).

### MICA-based biomarker discovery

**Figure 6:**
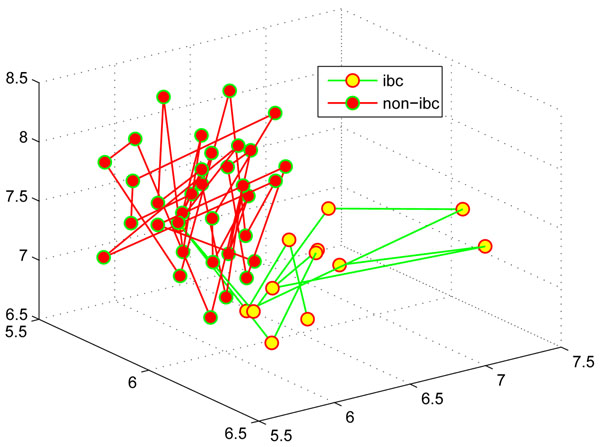
**Biomarker visualization in the stroma data**. Visualization of 47 samples in the stroma data by using three biomarkers

In addition to classifying large scale heterogeneous tumor profiles with exceptional performance, multi-resolution independent component analysis can be also applied to capture biomarkers for microarray profiles. We present a MICA-based filter-wrapper biomarker capturing algorithm and apply it to the stroma data. The details of this algorithm can be found in the Additional file 5. Table 4 lists the details on all the three biomarkers captured, where the SVM-rate for each biomarker is the classification ratio achieved by a SVM classifier with the ‘*rbf*’ kernel on the biomarker under leave-one-out cross validations. The order of the three biomarkers in Table 4 is listed according to its order identified in the biomarker discovery process. The SVM accuracy under the three biomarkers is 97.87% and the corresponding sensitivity and specificity are 92.31% and 100% respectively. The first biomarker is gene USP46, which is a broadly expressed gene reported as one gene associated with breast cancer and glioblastomas [18]. The second biomarker is FOSL2, which is one of four members in the *Fos* gene family. It is responsible for encoding leucine zipper proteins, which is able to dimerize with proteins of the *JUN* family, and form the transcription factor complex AP-1. As a regulator in cell proliferation, differentiation, and transformation, recent studies [19,20] have showed that it is one of important genes associated with breast cancer, by being involved in the regulation of breast cancer invasion and metastasis. The third biomarker is gene RPL5, which encodes a ribosomal protein that catalyzes protein synthesis. It was reported to associate with biosynthesis and energy utilization that is a cellular function associated with pathogenesis of breast cancer [21]. In addition, it also links to the breast cancer by lowering MDM2, which is a major regulator of p53 levels, preventing p53 ubiquitination and increasing its transcriptional activity [22]. Figure 6 visualizes the 47 samples (13 inflammatory breast cancers ('*ibc*') and 34 non-inflammatory breast cancers ('*non-ibc*')) of the stroma data using the three biomarkers. It is interesting to see that two types of cancers are separated into two spatially disjoint sets clearly, though one ‘ibc’ sample is wired in the ‘non-ibc’ samples.

**Table 4:** Three biomarkers discovered for the stroma data

Gene	Description	Bayes Factors	SVM-rates	MICA-coefficients
USP46	It belongs to a large family of cysteine proteases that function as deubiquitinating enzymes.	0.0093	0.8936	63.1453
FOSL2	It encodes leucine zipper proteins that can dimerize with proteins of the JUN family, thereby forming the transcription factor complex AP-1.	0.0418	0.8085	79.8313
RPL5	It encodes a ribosomal protein that catalyzes protein synthesis. It can lower MDM2 and prevent preventing p53 ubiquitination and increase its transcriptional activity.	0.5056	0.5957	81.8651

## Discussion

It is worthy to note that independent component analysis is a necessary step to achieve a good classification performance. A similar multi-resolution principal component analysis based SVM algorithm is not able to reach comparable performance as our algorithm because of the loss of statistical independence in the feature selection. Also, MICA-SVM encounters overfitting as SVM, PCA-SVM, ICA-SVM classifiers under the standard Gaussian kernel (‘*rbf*’), where each learning machine can only recognize the majority type samples of the training data in classification despite the testing sample type. Moreover, we have tried kernel ICA [23] based support vector machines (KICA-SVM) in our experiments in addition to the previous nine comparison algorithms. However, The KICA-SVM classifier generally has a lower performance level than the standard SVM classifier. Furthermore, the KICA-SVM not only shows a strong instability in classification but also inevitably encounters overfitting under the standard Gaussian kernel like the other learning machines. It seems to suggest that kernel based data reduction may not be a desirable approach in effective feature selection for high dimensional heterogeneous gene profiles. Similar results can be also found in kernel PCA [24] based support vector machine (KPCA-SVM) classifications: a KPCA-SVM classifier is essentially the PCA-SVM classifier when its two kernels are selected as ‘l*inear*’, otherwise, it encounters overfitting under the standard Gaussian kernel. In our ongoing project, in addition to further polishing our algorithm by comparing them with other state-of-the-art methods (e.g., SVM-RFE [2]), we are interested in theoretically validating the MICA-SVM‘s advantages over the classic SVM classifier from the viewpoint of Vapnik–Chervonenkis (VC) dimension theory [10].

## Conclusions

In this study, we present a novel multi-resolution feature selection algorithm: multi-resolution independent component analysis for effective feature selection for high-dimensional heterogeneous gene expression profiles, propose a high-performance MICA-SVM classification algorithm, and demonstrate its superiority and stability by comparing it with the nine state-of-the-art algorithms. Our algorithm not only consistently demonstrates the high-accuracy or clinical-level cancer diagnosis by treating an input profile a whole biomarker but also shows effectiveness in meaningful biomarker discovery. It suggests a great potential to facilitate high-throughput microarray technology into a clinical routine, especially, current classification methods have relative low even poor performance on the gene expression data. In addition, the multi-resolution data analysis based redundant global feature suppressing and effective local feature extraction will have a positive impact on large scale ‘*omics*’ data mining. In our future work, we plan to further explore MICA-SVM’s potential in other platform gene expression data, SNP, and protein expression data classification.

## Competing interests

The authors declare that they have no competing interests.

## Authors' contributions

HEY collects and processes the data, designs algorithms, implements the methods, and drafts paper. LXL participates in discussion and provides help to polish the paper. HEY and LXL jointly finalize the paper.

## Supplementary Material

Additional file 1 **Implementations of the four comparison algorithms** PCA-LDA, PCA-SVM, ICA-SVM, and NMF-SVM algorithm implementations Click here for file

Additional file 2 **MICA-LDA performance** MICA-LDA performance under 100 trials of 50% HOCV and 10-fold CV Click here for file

Additional file 3 **Comparing MICA-SVM with PLS-based regression methods** Comparisons MICA-SVM with three partial least square (PLS) based regression methods Click here for file

Additional file 4 Algorithmic stability analysis Click here for file

Additional file 5 MICA-based biomarker discovery algorithms Click here for file
