# Supplementary material for: Multi-resolution independent component analysis for high-performance tumor classification and biomarker discovery
Source: BMC Bioinformatics. 2011 Feb 15;12(Suppl 1):S7. doi: 10.1186/1471-2105-12-S1-S7 (PMC3044315; doi:10.1186/1471-2105-12-S1-S7)
Supplement: Additional file 1 — Implementations of the four comparison algorithms PCA-LDA, PCA-SVM, ICA-SVM, and NMF-SVM algorithm implementations [file 1471-2105-12-S1-S7-S1.pdf]

Supplemental Information (1)

Implementations of the four comparison algorithms: PCA-LDA, PCA-SVM, ICA-SVM and NMF-SVM

We have the following implementations about the four comparison algorithms. The PCA-LDA algorithm determines an unknown sample class type by employing linear discriminant analysis (LDA) in a subspace spanned by the principal components of the training data. The PCA-SVM (ICA-SVM) algorithm conducts the SVM classification by projecting the testing data into the subspace spanned by principal components (independent components) of the training data. The number of principal components in the PCA-LDA and PCA-SVM algorithms is selected such that their explained variance percentage (EVP) is 100%. The explained variance percentage (EVP) is the ratio between the accumulative variance from the selected data and the total data variance. For example, the explained variance percentage ρ_r from those first r principal components is defined as $\rho_r = \sum_{i=1}^{r} \lambda_i / \sum_{j=1}^{n} \lambda_j$, where λ_i is the data variance from the ith principal component. Alternatively, the number of independent components is selected as the number of input samples in the ICA-SVM algorithm. The NMF-SVM algorithm conducts the SVM classification for the meta-samples of input data computed through nonnegative matrix factorization (NMF). It is worthy to note that the cDNA data need to be converted to their corresponding nonnegative data before conducting the NMF-SVM classification. For a dataset $X \in \mathbb{R}^{p \times n}$ with negative entries, we simply convert it to a corresponding nonnegative matrix by $X^* = X + 2(\vec{1} \times |\theta|)$, where θ is the minimum negative entry in X and $\vec{1}$ is a $p \times n$ matrix with all '1' entries. Such a transform guarantees that the minimum entry in X^* is the absolute value of the minimum negative entry of X. Although another transform $X^* = X + (\vec{1} \times |\theta|)$ is also theoretically feasible, it may lead to X^* with many zeros for the 'breast_2' data and cause some convergence difficulties in nonnegative matrix factorizations. Since there is no optimal rank selection method available in NMF, we try matrix decomposition ranks from 2 to 10 in the NMF-SVM algorithm for all profiles. The final average classification rate is selected as the average classification rate at the rank where the NMF-SVM algorithm achieves the best performance.
