# Supplementary material for: Multi-resolution independent component analysis for high-performance tumor classification and biomarker discovery
Source: BMC Bioinformatics. 2011 Feb 15;12(Suppl 1):S7. doi: 10.1186/1471-2105-12-S1-S7 (PMC3044315; doi:10.1186/1471-2105-12-S1-S7)
Supplement: Additional file 2 — MICA-LDA performance MICA-LDA performance under 100 trials of 50% HOCV and 10-fold CV [file 1471-2105-12-S1-S7-S2.pdf]

# Supplemental Information (2)

## MICA-LDA algorithm performance on 100 trials of 50% HOCV and 10-fold CV

| Dataset | Avg. classification rate ± std (%) | Avg. sensitivity± std (%) | Avg. specificity ± std (%) |
|---|---|---|---|
| **50%HOCV** | | | |
| Stroma | 85.39±06.26 | 92.55±06.88 | 70.29±28.72 |
| Breast_1 | 97.64±01.50 | 98.03±01.88 | 96.57±03.74 |
| Prostate | 88.37±06.87 | 84.90±08.44 | 93.19±09.65 |
| Glioma | 95.00±11.25 | 93.33±21.08 | 96.67±10.54 |
| HCC | 84.17±09.25 | 88.23±11.44 | 76.76±17.52 |
| Breast_2 | 91.92±04.17 | 90.91±07.51 | 93.24±05.10 |
| **10-fold CV** | | | |
| Stroma | 87.50±14.39 | 89.17±18.45 | 85.00±33.75 |
| Breast_1 | 98.14±02.40 | 98.16±02.96 | 98.33±05.27 |
| Prostate | 93.52±06.26 | 89.82±07.99 | 98.33±05.27 |
| Glioma | 95.00±11.25 | 93.33±21.08 | 96.67±10.54 |
| HCC | 91.67±11.79 | 90.00±17.48 | 95.00±15.81 |
| Breast_2 | 93.78±05.37 | 96.00± 08.43 | 91.00±11.74 |
