# Supplementary material for: Multi-resolution independent component analysis for high-performance tumor classification and biomarker discovery
Source: BMC Bioinformatics. 2011 Feb 15;12(Suppl 1):S7. doi: 10.1186/1471-2105-12-S1-S7 (PMC3044315; doi:10.1186/1471-2105-12-S1-S7)
Supplement: Additional file 3 — Comparing MICA-SVM with PLS-based regression methods Comparisons MICA-SVM with three partial least square (PLS) based regression methods [file 1471-2105-12-S1-S7-S3.pdf]

Supplemental Information (3)

Comparing MICA-SVM with partial least square (PLS) based regression methods.

We also compare our algorithm with three PLS-based regression methods. As an important dimension reduction algorithm originally developed in the field of chemometrics, PLS recently draws more and more attention in gene expression data analysis. The three PLS-based regression methods consist of the PLS-based regression [1], PLS-based linear logistic regression proposed by Nguyen and Roche [2], and PLS-based ridge penalized logistic regression proposed by Fort and Lambert-Lacroix [3]. In our context, all the three algorithms treat classification as a regression one with discrete outputs under few observations and many predictor variables. We refer them as PLS-REG, NR-LLD, and RPLS-LLD respectively. Since the NR-LLD and RPLS-LLD algorithms require feature selection before classification, we conduct a two-sample t-test with pooled variance estimate to select the 2000 mostly differentiated expressed genes from each profile for the two methods. The number of PLS components are uniformly selected as 10 for all the three. The following Table shows the four algorithms' average classification rates and their standard deviations from the two cross validations. It is interesting to see that the MICA-SVM algorithm show strong advantages over the three peers in performance and stability. Moreover, it seems that the two-sample t-test based feature selection can help improve the NR-LLD, and RPLS-LLD‘s performance over the PLS-REG algorithm on the stroma and breast_2 data. But it is not always true for the other four data sets.

Table Performance of MICA-SVM, PLS-REG, NR-LLD, and RPLS-LLD algorithms

Algorithms	MICA-SVM	PLS-REG	NR-LLD	RPLS-LLD
**Data**	*Average classification rates under the 100 trials of 50% HOCV (%)*			
Stroma	98.26±02.25	71.57±06.87	83.81±05.58	85.52±07.05
Breast_1	99.04±00.99	86.16±03.00	84.79±03.13	86.66±02.81
Prostate	99.69±00.67	90.87±02.65	89.22±03.25	89.21±03.19
Glioma	98.76±02.03	73.52±07.62	69.28±08.31	69.36±08 .38
HCC	98.30±02.30	60.38±07.90	60.43±08.68	60.43±08.85
Breast_2	97.23±03.20	62.23±05.65	80.87±05.81	81.02±05.76
	Average classification rates under 10-fold CV (%)			
Stroma	98.00±06.32	79.00±13.50	91.50±11.07	91.50±11 .07
Breast_1	99.52±01.51	87.01±04.81	86.08±04.94	88.38±05.10
Prostate	100.0±00.00	91.98±07.22	92.64±05.99	94.12±04.58
Glioma	100.0±00.00	75.00±22.46	69.33±25.76	73.00±26 .46
HCC	100.0±00.00	65.00±09.46	70.00±15.32	70.00±15.32
Breast_2	99.00±03.16	70.11±14.05	87.67±12.46	86.78±13.33

References

1. Wold, S., et al (2001) PLS-regression: a basic tool of chemometrics, *Chemometrics and Intelligent Laboratory Systems* Vol 58 (2), 109-130
2. Nguyen, D. and Rocke, D. (2002) Tumor classification by partial least squares using microarray gene expression data. *Bioinformatics*. 18. 39–50
3. Fort, G. and Lambert-Lacroix S. (2005) Classification using partial least squares with penalized logistic regression, *Bioinformatics*, 21(7):1104-1111
