# Supplementary material for: Multi-resolution independent component analysis for high-performance tumor classification and biomarker discovery
Source: BMC Bioinformatics. 2011 Feb 15;12(Suppl 1):S7. doi: 10.1186/1471-2105-12-S1-S7 (PMC3044315; doi:10.1186/1471-2105-12-S1-S7)
Supplement: Additional file 4 — Algorithmic stability analysis [file 1471-2105-12-S1-S7-S4.pdf]

Supplemental Information (4)

Algorithmic stability analysis

The instabilities of current classification methodologies are widely found in gene expression analysis, especially for the algorithms developed for individual data, i.e., some algorithms can only work well for one or very few specific data sets and show very high instability in classification when applied to the other data. For example, in our experiment, SVM achieves 91.16% and 86.40% average classification rates on the 'prostate' and 'breast_1' data under the 100 trials of 50% HOCV, but it can only achieve 61.93% and 63.04% average classification rates on the 'HCC' and 'breast_2' data under the same cross validation. The instabilities not only present difficulties in reproducible biomarker discovery, but also hamper exploring its clinical potential. However, there is even no ad-hoc investigation on algorithmic stability analysis. To evaluate the algorithmic stabilities of gene expression classification algorithms, we present an algorithmic stability analysis by introducing two scale-free measures: algorithm stability index and relative stability. The algorithm stability index measures the stability of an algorithm across a number of data sets, which can be heterogeneous data sets generated from different microarray profiling platforms or processed by different pre-processing methods. A high algorithm index value indicates better stability of an algorithm. Alternatively, the relative stability measures the stabilities of a set of classification algorithms with respect to a specific algorithm, which is selected as MICA-SVM for its outstanding performance. A small relative stability indicates an algorithm has a relatively close performance to MICA-SVM. Given a classification algorithm running on M heterogeneous profiles under a cross validation, the algorithm stability index δ_a and the relative stability δ_r are defined as, $\delta_a = \frac{1}{M}\sum_{i=1}^{M}(1-\frac{s_i}{\mu_i})$, $\delta_r = \frac{1}{M}\sum_{i=1}^{M}\frac{\mu_i^* - \mu_i}{s_i}$, where μ_i, s_i are the average classification rate and the corresponding standard deviation of the algorithm on the ith profile respectively, and the parameter μ_i^* is the average classification ratio of the MICA-SVM algorithm on the ith profile.

The two sub-figures in the following Figure show the algorithm stability index and relative algorithm stability values of all the seven algorithms on the six heterogeneous profiles under the 100 trials of 50% HOCV (LDA is excluded for its relatively low performance). It is interesting to see that the SVM, PCA-SVM, NMF-SVM, and PCA-LDA algorithms have almost same level stabilities for their close δ_a values. The smallest δ_a value suggests the least stabilities of the ICA-SVM algorithm. This is possibly because almost all independent components in the classic ICA are calculated from the global features and a large amount of redundant global features may get involved in the learning machine training. Finally, the SVM classifier would lose generality and show a high-level instability in performance. The δ_a values of MICA-SVM and MICA-LDA are the largest and 2nd largest among the seven algorithm index values. The relative stability value of MICA-LDA suggests it achieve the closest performance with respect to the MICA-SVM algorithm.

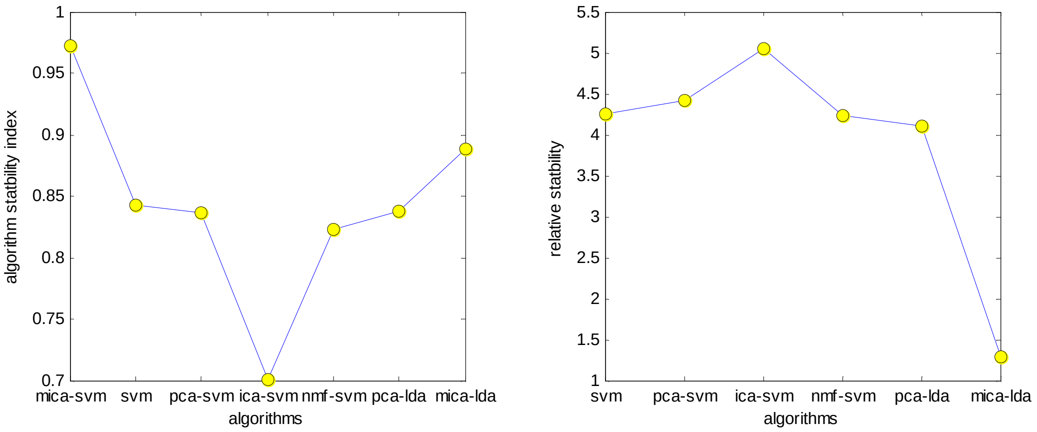

Figure The algorithm stability index and relative stability values under the 100 trials of 50% HOCV. MICA-SVM has the largest stability among all seven algorithms, and MICA-LDA has the closest performance to MICA-SVM
