# Supplementary material for: Multi-resolution independent component analysis for high-performance tumor classification and biomarker discovery
Source: BMC Bioinformatics. 2011 Feb 15;12(Suppl 1):S7. doi: 10.1186/1471-2105-12-S1-S7 (PMC3044315; doi:10.1186/1471-2105-12-S1-S7)
Supplement: Additional file 5 — MICA-based biomarker discovery algorithms [file 1471-2105-12-S1-S7-S5.pdf]

Supplemental Information (5)

MICA-based biomarker discovery

We present a MICA-based filter-wrapper biomarker capturing algorithm and apply it to two benchmark profiles. In this algorithm, MICA and the Bayesian two-sample t-test, which has been proved to be superior to the classic t-test for microarray data [1], are employed in filtering to screen biomarker candidates. Alternatively, SVM classifier with a '*rbf*' kernel under a leave-one-out cross validation (LOOCV) works as a wrapper to collect biomarkers. The multi-resolution independent component analysis based biomarker discovery approach can be described as follows.

Given an input dataset $X \in \mathbb{R}^{m \times n}$ with n samples across m genes, we firstly filter a potential biomarker set S_b by conducting the two-sample Bayesian t-test to evaluate genes according to their differentially expressed levels. The potential biomarker set S_b consists of significantly differentially-expressed genes. For each dataset, we select a number of genes with the smallest Bayesian factors: $|S_b| = \max\{\lceil m \times 0.01 \rceil, 200\}$ to construct S_b. Then, multi-resolution independent component analysis is employed to conduct the decomposition: $X \sim AZ$, where $A \in \mathbb{R}^{n \times k}$ is the mixing matrix and Z is the independent component matrix: $Z = [z_1, z_2 \cdots z_k]$, $z_i \in \mathbb{R}^{m \times 1}$. For each gene, a coefficient η is used to rank its contribution to all ICs. For example, the coefficient for the ith gene is calculated as $\eta_i = \|z_i\|_2$. A large coefficient value of a gene indicates that it has significant contributions to the ICs.

Each gene in S_b is used to train the SVM classifier under LOOCV. The first biomarker g_1 is selected as the gene with the highest accuracy. If there is more than one candidate, the gene with the largest coefficient in the MICA-ranking will be selected. The potential biomarker set is updated by removing the selected biomarker, i.e., $S_b = S_b - \{g_1\}$. The second biomarker gene g_2 is selected from the current potential-biomarker set S_b such that the SVM classifier reaches its maximum classification rate for the combination of g_1 and g_2. If there are more than one candidate, the gene with the largest coefficient value ranked by MICA will be selected as g_2. Similarly, S_b is updated $S_b = S_b - \{g_2\}$. Such a proceeding continues until the SVM classifier achieves the maximum classification accuracy with the fewest biomarkers.

In addition to the stroma data, we also apply our biomarker discovery algorithm to another benchmark profile: medulloblastoma data [2], and capture two biomarkers: NDP (X65724) and RPL21 (U25789). The total SVM accuracy under the two biomarkers achieves 97.06% with 100.0% specificity and 88.89% sensitivity. The first biomarker is NDP, a gene related to Norrie disease. The Norrie disease is reported as a rare genetic disorder characterized by bilateral congenital blindness, caused by a vascularized mass behind each lens due to pseudoglioma [3]. This finding strongly suggests that the medulloblastoma has very similar phenotypes as the glioma and there are some genes related to both cancers. Interestingly, the medulloblastoma was originally considered as a glioma [4]. The second biomarker is RPL21, a gene encoding ribosomal proteins and has multiple processed pseudogenes dispersed through the genome. It was reported as one of biomarkers related to brain and other CNS cancers [3,5]. The results show that our proposed method is able to discover the biologically meaningful knowledge.

References

1. Fox, R. and Dimmic, M.: A two-sample Bayesian t-test for microarray data, *BMC Bioinformatics* 7(126) (2006)
2. Brunet, J., Tamayo, P., Golub, T. and Mesirov, J.: Molecular pattern discovery using matrix factorization," *Proc. Natl Acad. Sci. USA* 101(12), 4164–4169, 2004.
3. Stein, A. Litman, T., Fojo, T. and Bates, S.: A Serial Analysis of Gene Expression (SAGE) Database Analysis of Chemosensitivity: Comparing Solid Tumors with Cell Lines and Comparing Solid Tumors from Different Tissue Origins. Cancer Research 64, 2805–2816 (2004)
4. Jallo, G.: Medulloblastoma, *eMedicine* (2007)
5. Pomeroy, S.L., et al (2002). Prediction of central nervous system embryonal tumour outcome based on gene expression. *Nature*, 415(6870), 436-442.
